# A dosimetric comparison of volumetric modulated arc therapy (VMAT) and non-coplanar intensity modulated radiotherapy (IMRT) for nasal cavity and paranasal sinus cancer

**DOI:** 10.1186/1748-717X-9-193

**Published:** 2014-08-30

**Authors:** Yuri Jeong, Sang-wook Lee, Jungwon Kwak, Ilsung Cho, Sang Min Yoon, Jong Hoon Kim, Jin-hong Park, Eun Kyung Choi, Si Yeol Song, Young Seok Kim, Su Ssan Kim, Ji Hyeon Joo, Seung Do Ahn

**Affiliations:** Department of Radiation Oncology, Asan Medical Center, University of Ulsan College of Medicine, 88, Olympic-ro-43-gil, Songpa-gu, Seoul, 138-736 Republic of Korea

**Keywords:** Paranasal sinus cancer, Nasal cavity cancer, Volumetric modulated arc therapy, Non-coplanar intensity modulated radiotherapy, Planning target volume, Organs at risk

## Abstract

**Background:**

To compare dosimetric parameters of volumetric modulated arc therapy (VMAT) and non-coplanar intensity modulated radiotherapy (IMRT) for nasal cavity and paranasal sinus cancer with regard to the coverage of planning target volume (PTV) and the sparing of organs at risk (OAR).

**Methods:**

Ten patients with nasal cavity or paranasal sinus cancer were re-planned by VMAT (two-arc) plan and non-coplanar IMRT (7-, 11-, and 15-beam) plans. Planning objectives were to deliver 60 Gy in 30 fractions to 95% of PTV, with maximum doses (D_max_) of <50 Gy to the optic nerves, optic chiasm, and brainstem, <40 Gy to the eyes and <10 Gy to the lenses. The target mean dose (D_mean_) to the parotid glands was <25 Gy, and no constraints were applied to the lacrimal glands. Planning was optimized to minimized doses to OAR without compromising coverage of the PTV. VMAT and three non-coplanar IMRT (7-, 11-, and 15-beam) plans were compared using the heterogeneity and conformity indices (HI and CI) of the PTV, D_max_ and D_mean_ of the OAR, treatment delivery time, and monitor units (MUs).

**Results:**

The HI and CI of VMAT plan were superior to those of the 7-, 11-, and 15-beam non-coplanar IMRT. VMAT and non-coplanar IMRT (7-, 11-, and 15-beam) showed equivalent sparing effects for the optic nerves, optic chiasm, brainstem, and parotid glands. For the eyes and lenses, VMAT achieved equivalent or better sparing effects when compared with the non-coplanar IMRT plans. VMAT showed lower MUs and reduced treatment delivery time when compared with non-coplanar IMRT.

**Conclusions:**

In 10 patients with nasal cavity or paranasal sinus cancer, a VMAT plan provided better homogeneity and conformity for PTV than non-coplanar IMRT plans, with a shorter treatment delivery time, while achieving equal or better OAR-sparing effects and using fewer MUs.

## Background

Nasal cavity and paranasal sinus cancers are relatively rare and account for about 5% of head and neck cancers [1, 2]. Most patients are diagnosed at an advanced stage, because symptoms and signs are usually nonspecific or asymptomatic in earlier stages. Standard treatment for locally advanced nasal cavity and paranasal sinus cancer is surgery followed by postoperative radiotherapy, or definitive radiotherapy with or without chemotherapy. In locally advanced stage, complete resection may not be possible due to the proximity to critical organs, and postoperative or definitive radiotherapy is needed. However, it is challenging to obtain optimal dose coverage for the target volume without compromising critical organs due to the proximity of critical organs such as eyes, optic nerves, optic chiasm, and brain. Intensity-modulated radiotherapy (IMRT) can provide more conformal dose coverage for the target volume, with reduced dosage to the adjacent critical organs, when compared with two- or three-dimensional radiotherapy [3–7]. Moreover, non-coplanar beams may provide additional optimization capacity within inverse planning processes for the target volumes of the nasal cavity and paranasal sinus cancer, which are usually located between both eyes. Recently, volumetric modulated arc therapy (VMAT) delivered by linear accelerator (LINAC) has become available. VMAT may reduce treatment delivery time with lower monitor units (MUs) while providing conformal dose distribution [8–11].

This study compares VMAT and non-coplanar IMRT for nasal cavity and paranasal sinus cancers with regard to the coverage of planning target volume (PTV) and the sparing of organs at risk (OAR).

## Methods

### Patients and target volume

Ten patients with nasal cavity or paranasal sinus cancers, who were previously treated with three-dimensional radiotherapy or IMRT between April 2011 and April 2013, were randomly selected from a clinical database maintained by the Asan Medical Center, Seoul, Korea. Treatment with VMAT (two-arc) and non-coplanar IMRT (7-, 11-, and 15-beam) was re-planned. Patient characteristics are shown in Table 1. All patients were T3-4N0M0 according to the American Joint Committee on Cancer 7th staging. Each patient was simulated with a thermoplastic mask to immobilize the head and neck. Computed tomography (CT) images with a 2.5 mm slice thickness were obtained from vertex to clavicle. For patients previously treated with definitive radiotherapy, gross tumor volume (GTV) was defined as all gross disease on the magnetic resonance imaging (MRI), CT, or positron emission tomography (PET). Clinical target volumes (CTVs) included the nasal cavity, ethmoid sinus, frontal sinus, or maxillary sinus depending on the tumor location and the extent to cover microscopic spread (Table 1). To compensate for daily set-up variation and motion, the PTV was defined as the CTV plus a 5 mm margin. Contoured OAR were the eyes, lenses, optic nerves, optic chiasm, lacrimal glands, brainstem, brain, and parotid glands.

**Table 1:** **Patient characteristics**

Case	Primary site	Stage ^a^	Aim of radiotherapy	Clinical target volume
1	Nasal cavity	T4aN0M0	Definitive	Nasal cavity, ethmoid, frontal sinus, ipsilateral maxillary sinus
2	Nasal cavity	T4aN0M0	Definitive	Nasal cavity, ethmoid, frontal sinus, ipsilateral maxillary sinus
3	Maxillary sinus	T4aN0M0	Definitive	Nasal cavity, ipsilateral maxillary sinus
4	Maxillary sinus	T4aN0M0	Postoperative	Nasal cavity, ethmoid, frontal sinus, ipsilateral maxillary sinus
5	Nasal cavity	T4aN0M0	Postoperative	Nasal cavity, ethmoid, frontal sinus, ipsilateral maxillary sinus
6	Ethmoid	T4bN0M0	Postoperative	Nasal cavity, ethmoid, frontal sinus, bilateral maxillary sinus
7	Maxillary sinus	T3N0M0	Postoperative	Nasal cavity, ipsilateral maxillary sinus
8	Nasal cavity	T4aN0M0	Definitive	Nasal cavity, ethmoid, frontal sinus, ipsilateral maxillary sinus
9	Nasal cavity	T3N0M0	Postoperative	Nasal cavity, ethmoid, frontal sinus, bilateral maxillary sinus
10	Nasal cavity	T4bN0M0	Postoperative	Nasal cavity, ethmoid, frontal sinus, ipsilateral maxillary sinus

^a^According to American Joint Committee on Cancer 7th staging.

### Planning objectives

The planning objectives were the same for VMAT and non-coplanar IMRT. The first objective was to deliver a prescribed dose of 60 Gy in 30 fractions to at least 95% of the PTV. Second, both treatments were planned with a maximum dose (D_max_) of <50 Gy to the optic nerves, optic chiasm, and brainstem, <40 Gy to the eyes and <10 Gy to the lenses. The planned mean dose (D_mean_) to the parotid glands was < 25 Gy, and no constraint was applied to the lacrimal glands. Third, treatment planning aimed to reduce doses to OAR as much as possible without compromising the coverage of the PTV.

Priority of optimization was same as the order of planning objectives mentioned above. We put the highest priority to the maximum and minimum doses of PTV. For OARs, we put higher priority to the maximum doses of optic nerves, eyes, and lenses because these organs were very close to the PVT. After optimization, actual dose distributions in PTV and OARs were checked by dose statistics, dose volume histograms, and 3-dimensional isodose lines whether planning objectives were met. If the optimization result was not acceptable, we modified optimization parameter such as priority and dose constraint and performed optimization again.

### VMAT planning

For both VMAT and non-coplanar IMRT plans, 6 MV photon beams were applied using a Varian TrueBeam STx (Varian Medical System, Palo Alto, CA), with multi-leaf collimators (MLCs) comprising 120 leaves of 2.5 mm width (in sliding window mode). Dose calculations were performed by Anisotropic Analytic Algorithm with a maximum dose rate of 600 MU/min. VMAT plans were created using the two-arc technique, and optimization was performed using Eclipse progressive resolution optimization (version 10.0.28). The first arc ranged from 181° to 179° in clockwise rotation, and the second arc was from 179° to 181° in counter-clockwise rotation. The collimator angle was 30° for the first arc and 330° for the second arc.

### IMRT planning

Eclipse dose volume optimization (version 10.0.28) was used for IMRT planning. To estimate the optimal number and angles of beams in non-coplanar IMRT plans for nasal cavity and paranasal sinus cancers, a beam angle optimization process was performed for 5 patients as an experimental purpose. The beam angle optimization was automatic process which is supported by Eclipse dose volume optimization (version 10.0.28). The optimal number of beams varied from 5 to 15 in each patient, and the beams were concentrated in the anterior direction, sparing both eyes and lenses. Based on these results, three non-coplanar IMRT (7-, 11-, and 15-beam) plans were developed for each patient. The 7-beam IMRT consisted of 5 coplanar beams at gantry angles of 0°, 30°, 100°, 260°, and 330°, and 2 non-coplanar vertex beams at gantry angles of 30° and 330° with a 90° couch rotation. The collimator angle was 30° for the 2 coplanar beams at gantry angles of 30° and 100°, 330° for the 2 coplanar beams at gantry angles of 260° and 330°, and 0° for the other beams. The 11-beam IMRT used 9 coplanar beams at gantry angles of 0°, 20°, 40°, 60°, 100°, 260°, 300°, 320°, and 340°, and 2 non-coplanar vertex beams at gantry angles of 30° and 330° with a 90° couch rotation. The collimator angle was 30° for the 4 coplanar beams at gantry angles between 20° and 100°, 330° for the 4 coplanar beams at gantry angles between 260° and 340°, and 0° for other beams. The 15-beam IMRT comprised 11 coplanar beams at gantry angles of 0°, 15°, 30°, 45°, 60°, 100°, 260°, 300°, 315°, 330° and 345°, and 4 non-coplanar vertex beams at gantry angles of 15°, 30°, 330° and 345° with a 90° couch rotation. The collimator angle was 30° for the 5 coplanar beams at gantry angles between 15° and 100°, 330° for the 5 coplanar beams at gantry angles between 260° and 345°, and 0° for other beams.

### Plan evaluation and statistics

PTV coverage was compared between VMAT and non-coplanar IMRT (7-, 11-, and 15-beam) using the heterogeneity index (HI) and conformity index (CI). The HI of PTV was defined as:

HI = (D_5%_ - D_95%_)/D_mean_

D_5%_ and D_95%_ are the minimum doses delivered to 5% and 95% volume of the PTV, and D_mean_ is the mean dose. A greater HI means higher heterogeneity. The CI of PTV was defined as:

CI = V_TV_/V_PTV_

where V_TV_ is the treatment volume enclosed by the prescribed 60 Gy isodose surface, and V_PTV_ is the volume of the PTV. A greater CI indicates lower conformity. To compare the OAR sparing of the VMAT and each of non-coplanar IMRT plans, D_max_ was evaluated for the eyes, lenses, optic nerves, optic chiasm, lacrimal glands, and the brainstem, and the D_mean_ of the parotid glands was recorded. Mean dose volume histograms (DVHs) for the PTV and OAR were calculated. MUs and treatment time for 2 Gy were also evaluated. We defined treatment delivery time in each patient as the sum of the beam on time with gantry rotation and beam setup time for VMAT plans and as the sum of the beam on time, beam setup time, and couch rotation time for non-coplanar IMRT plans, respectively. To estimate real treatment delivery time consisting of beam on time, beam setup time, and couch rotation time, VMAT and non-coplanar IMRT plans were delivered on Varian TrueBeam STx in quality assurance mode. Wilcoxon signed-rank tests were performed to compare the above parameters between the VMAT plan and each of the non-coplanar IMRT (7-, 11-, and 15-beam) plans. All statistical tests were two-sided and performed at the 5% level of significance using SPSS (version 18.0).

## Results

### Planning target volume coverage

**Figure 1:**
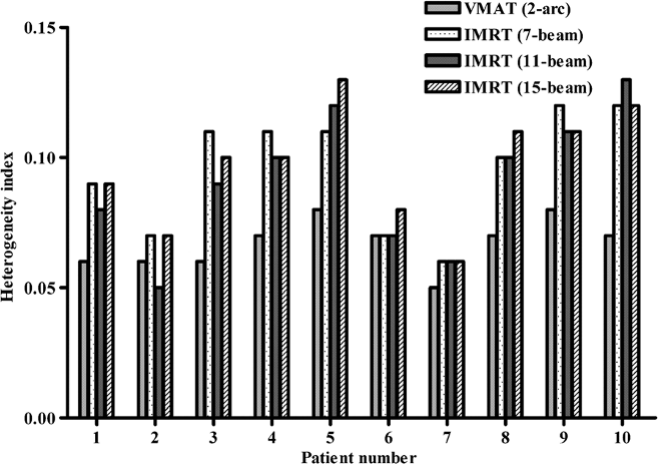
**Comparison of the heterogeneity indices of volumetric modulated arc therapy (VMAT) and non-coplanar intensity modulated radiotherapy (IMRT; 7-, 11-, and 15-beam).**

**Figure 2:**
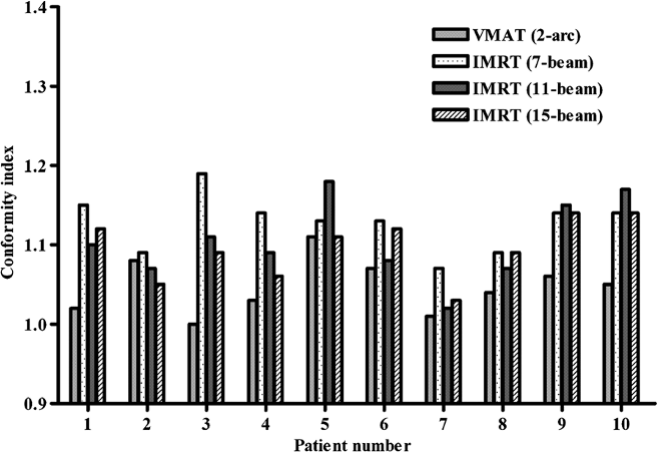
**Comparison of the conformity indexes of volumetric modulated arc therapy (VMAT) and non-coplanar intensity modulated radiotherapy (IMRT; 7-, 11-, and 15-beam).**

**Figure 3:**
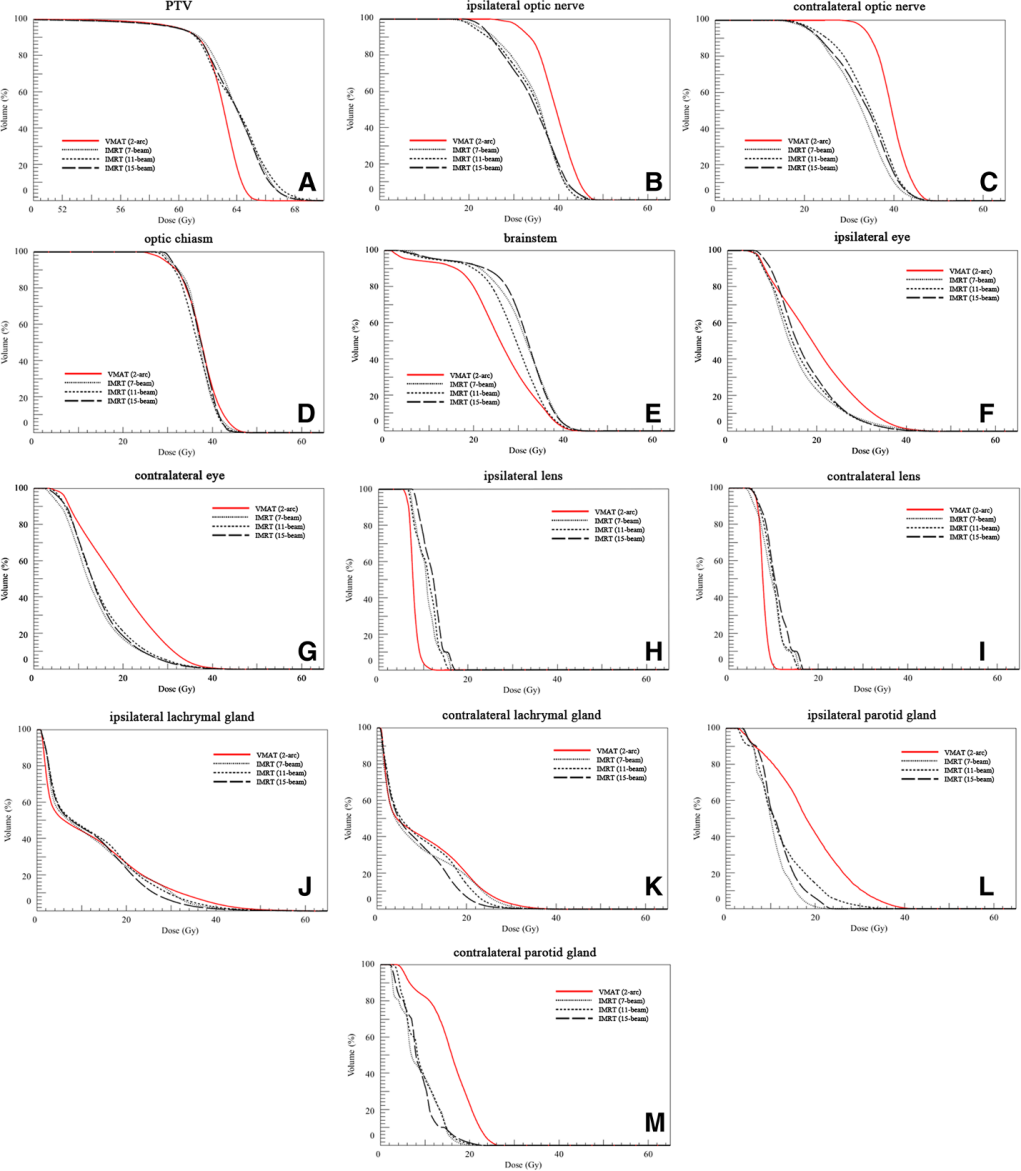
**Mean dose volume histograms (DVHs) for planning target volume (PTV) and organs at risk in volumetric modulated arc therapy (VMAT) and non-coplanar intensity modulated radiotherapy (IMRT) using 7-, 11-, and 15-beams. (A)** PTV; **(B)** ipsilateral optic nerve; **(C)** contralateral optic nerve; **(D)** optic chiasm; **(E)** brainstem; **(F)** ipsilateral eye; **(G)** contralateral eye; **(H)** ipsilateral lens; **(I)** contralateral lens; **(J)** ipsilateral lachrymal gland; **(K)** contralateral lachrymal gland; **(L)** ipsilateral parotid gland; and **(M)** contralateral parotid gland.

The HI and CI of VMAT and each of the 7-, 11-, and 15-beam non-coplanar IMRT plans for each of the 10 patients are shown in Figures 1 and 2. The HIs of the VMAT plan were better than those of the 7-, 11-, and 15-beam non-coplanar IMRT plans (mean HI 0.07 vs. 0.10 vs. 0.09 and 0.10, respectively, p = 0.004, p = 0.012, and p = 0.005; Table 2). The CI was also superior in VMAT plans compared with each of 7-, 11-, 15-beam non-coplanar IMRT plans (mean CI, 1.05 vs. 1.13 vs. 1.10 and 1.10, respectively, p = 0.002, p = 0.008, and p = 0.016) (Table 2). The mean DVHs for PTVs are shown in Figure 3.

**Table 2:** **Dosimetric comparison for PTV, OARs, and MUs between VMAT and non-coplanar IMRT using 7-, 11-, and 15-beam**

	VMAT Mean (SD)	7-beam IMRT Mean (SD)	11-beam IMRT Mean (SD)	15-beam IMRT Mean (SD)	***p*** value (VMAT vs.)		
					7-beam IMRT	11-beam IMRT	15-beam IMRT
HI of PTV	0.07 (0.01)	0.10 (0.03)	0.09 (0.03)	0.10 (0.02)	0.004*	0.012*	0.005*
CI of PTV	1.05 (0.03)	1.13 (0.03)	1.10 (0.05)	1.10 (0.04)	0.002*	0.008*	0.016*
Ipsilateral optic nerve D_max_ (Gy)	47.9 (2.16)	46.9 (3.15)	46.3 (3.21)	46.0 (3.91)	0.492	0.492	0.193
D_1%_ (Gy)	45.7 (2.36)	44.1 (2.95)	43.0 (2.40)	43.1 (3.17)	0.322	0.084	0.064
Contralateral optic nerve D_max_ (Gy)	47.0 (2.86)	46.0 (2.86)	46.8 (2.79)	46.0 (2.87)	0.275	0.695	0.322
D_1%_ (Gy)	45.3 (2.65)	43.1 (2.79)	44.2 (2.21)	43.6 (2.55)	0.129	0.275	1.000
Optic chiasm D_max_ (Gy)	46.7 (2.64)	45.3 (3.46)	45.6 (2.67)	45.0 (2.87)	0.275	0.322	0.193
D_1%_ (Gy)	46.6 (2.48)	42.7 (3.01)	42.9 (2.45)	42.5 (2.96)	0.193	0.166	0.131
Brainstem D_max_ (Gy)	44.2 (3.31)	46.0 (2.33)	44.5 (2.45)	45.1 (2.24)	0.105	0.770	0.322
D_1%_ (Gy)	39.8 (3.05)	41.7 (1.77)	40.9 (1.77)	41.8 (1.62)	0.160	0.359	0.064
Ipsilateral eye D_max_ (Gy)	43.1 (3.56)	48.8 (4.51)	46.1 (4.59)	45.9 (3.30)	0.004*	0.064	0.037*
D_1%_ (Gy)	38.8 (3.45)	39.8 (3.89)	37.6 (3.36)	37.4 (2.56)	0.322	0.322	0.275
Contralateral eye D_max_ (Gy)	41.1 (3.15)	44.7 (2.94)	42.7 (4.37)	42.4 (3.86)	0.004*	0.492	0.322
D_1%_ (Gy)	36.7 (3.70)	35.7 (2.89)	34.4 (4.03)	33.9 (3.84)	0.264	0.078	0.064
Ipsilateral lens D_max_ (Gy)	10.5 (1.36)	12.1 (2.28)	12.6 (1.99)	13.6 (1.79)	0.193	0.020*	0.002*
D_1%_ (Gy)	9.77 (1.19)	11.9 (2.32)	12.2 (2.10)	13.3 (1.96)	0.014*	0.006*	0.002*
Contralateral lens D_max_ (Gy)	10.2 (1.17)	11.6 (2.18)	11.8 (2.19)	12.6 (2.12)	0.193	0.084	0.006*
D_1%_ (Gy)	9.49 (0.93)	11.2 (2.36)	11.4 (2.26)	12.2 (2.29)	0.053	0.012*	0.04*
Ipsilateral lacrimal gland D_max_ (Gy)	28.5 (10.3)	15.2 (6.37)	18.1 (9.93)	16.5 (6.27)	0.004*	0.004*	0.002*
D_1%_ (Gy)	27.1 (1.02)	14.1 (5.50)	17.0 (9.41)	15.4 (5.71)	0.002*	0.004*	0.002*
Contralateral lacrimal gland D_max_ (Gy)	22.1 (4.44)	11.4 (6.06)	12.4 (6.02)	11.0 (5.27)	0.002*	0.002*	0.002*
D_1%_ (Gy)	21.0 (4.69)	10.8 (5.77)	11.3 (5.74)	10.4 (5.17)	0.002*	0.002*	0.002*
Ipsilateral parotid gland D_mean_ (Gy)	12.5 (9.91)	12.2 (8.75)	12.7 (8.93)	11.8 (8.45)	0.922	0.625	0.625
Contralateral parotid gland D_mean_ (Gy)	9.84 (7.80)	8.84 (6.87)	9.27 (6.80)	8.07 (5.87)	0.131	0.492	0.084
Monitor units	485 (66.3)	2224 (423)	2350 (451)	2475 (384)	0.002*	0.002*	0.002*
Treatment delivery time (Minutes)	4.34 (0.00)	11.72 (0.71)	15.65 (0.75)	19.58 (0.64)	0.002*	0.002*	0.002*

*p value < 0.05.

PTV, planning target volume; OARs, organs at risk; MUs, monitor units; SD, standard deviation; HI, heterogeneity index; CI, conformity index; D_max_, maximum dose; D_mean_, mean dose; D_1%_, minimum dose delivered 1% of the PTV.

### Dose to organs at risk

**Figure 4:**
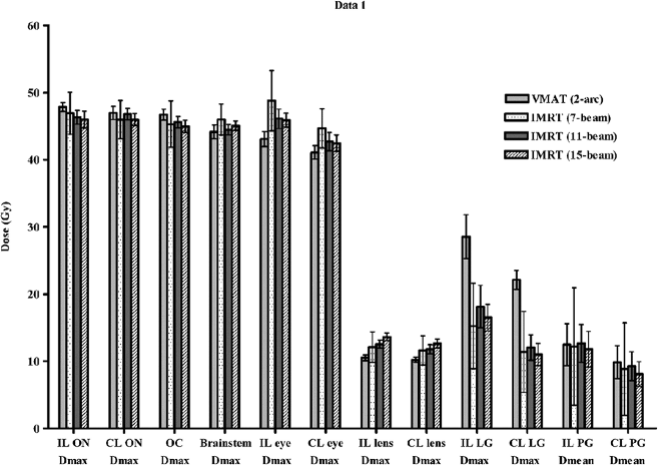
**Comparison of dose distribution in organs at risk for volumetric modulated arc therapy (VMAT) and non-coplanar intensity modulated radiotherapy (IMRT; 7-, 11-, and 15-beam).** IL, ipsilateral; CL, contralateral; D_max_, maximum dose; D_mean_, mean dose; ON, optic nerve; OC, optic chiasm; LG, lachrymal gland; PG, parotid gland.

The doses delivered to the various OAR by the VMAT plan and each of the 7-, 11-, and 15-beam non-coplanar IMRT plans for each of 10 patients are shown in Figure 4. The VMAT plan and the non-coplanar IMRT (7-, 11-, and 15-beam) plans showed equivalent sparing effects for the optic nerves, optic chiasm, brainstem, and parotid glands. For the eyes and lenses, VMAT achieved better or equivalent sparing effects when compared with each of the non-coplanar IMRT plans (Table 2). The maximum doses to the eyes of the VMAT plan were equivalent to those of the 11-beam non-coplanar IMRT plan (mean D_max_ of ipsilateral eye, 43.1 Gy vs. 46.1 Gy, p = 0.064; mean D_max_ of contralateral eye, 41.1 Gy vs. 42.7 Gy, p = 0.492), but lower than those of the 7-beam non-coplanar IMRT plan (mean D_max_ of ipsilateral eye, 43.1 Gy vs. 48.8 Gy, p = 0.004; mean D_max_ of contralateral eye, 41.1 Gy vs. 44.7 Gy, p = 0.004). For lenses, VMAT had an equivalent sparing effect to the 7-beam non-coplanar IMRT plan (mean D_max_ of ipsilateral lens, 10.5 Gy vs. 12.1 Gy, p = 0.193; mean D_max_ of contralateral lens, 10.2 Gy vs. 11.6 Gy, p = 0.193) and a better sparing effect than 15-beam non-coplanar IMRT plan (mean D_max_ of ipsilateral lens, 10.5 Gy vs. 13.6 Gy, p = 0.002; mean D_max_ of contralateral lens, 10.2 Gy vs. 12.6 Gy, p = 0.006). For lacrimal glands which no constraints were applied during treatment planning, each of the non-coplanar IMRT plans showed better D_max_ and D_1%_ than VMAT (Table 2). The mean DVHs for OAR are shown in Figure 3. Although DVHs for the optic chiasm, lenses, and lacrimal glands showed a similar trend to the D_max_, the DVHs for the optic nerves and eyes were better in non-coplanar IMRT, and the DVHs for brainstems were better in VMAT.

### MUs and treatment delivery times

Mean MU was significantly lower in the VMAT plan than for each of 7-, 11-, 15-beam non-coplanar IMRT plans (485 vs. 2224 vs. 2350 and 2475, respectively; p = 0.002, p = 0.002, and p = 0.002). The mean beam on time was shorter in the VMAT plan than in the 7-, 11-, and 15-beam non-coplanar IMRT plans (2.48 minutes vs. 3.71 minutes vs. 3.92 minutes and 4.13 minutes, respectively; p = 0.002, p = 0.002, and p = 0.002). The estimated beam setup time was 1.86 minutes in VMAT plans and 6.51 minutes, 10.23 minutes, and 13.95 minutes in each of 7-, 11-, and 15-beam non-coplanar IMRT plans, respectively. In non-coplanar IMRT plans, the couch rotation time was estimated as 1.5 minutes. The entire treatment delivery time was estimated shorter in VMAT plan than in the 7-, 11-, and 15-beam non-coplanar IMRT plans (mean, 4.34 minutes vs. 11.72 minutes vs. 15.65 minutes and 19.58 minutes, respectively; p = 0.002, p = 0.002, and p = 0.002) (Table 2).

## Discussion

In this study, the VMAT plan had better conformity and homogeneity with the PTV and equivalent to better sparing effects on OAR than the non-coplanar IMRT plans (7-, 11-, and 15- beam), for radiotherapy of nasal cavity and paranasal sinus cancers. The nasal cavity and paranasal sinus are adjacent to critical organs such as the eyes, optic nerves, optic chiasm, and brain. Optimizing dose coverage for the target volume without compromising critical organs is therefore a challenge in treatment planning. Previously, two-dimensional radiotherapy (2D-RT) with a weighted anterior field and two wedged lateral fields, and three-dimensional conformal radiotherapy (3D-CRT) were used to avoid both eyes and to overcome dose heterogeneity. However, the complex shape and tissue heterogeneity of the target volume negatively affect dose distribution, and treatment outcomes were unsatisfactory in terms of local control and optic pathway preservation [7]. With the development of computerized optimization processes, techniques with more conformal and homogeneous dose distributions have become possible. These include IMRT, a form of 3D-CRT able to generate non-uniform intensity using beamlets. Several studies have compared IMRT to 2D-RT or 3D-CRT for the treatment of nasal cavity and paranasal sinus cancer [3–7]. Lee et al. compared IMRT using the complementary boost-fields with 3D-CRT for ethmoid sinus cancer [4]. The homogeneity of the PTV was higher for IMRT than for 3D-CRT, and OAR sparing was similar. Other studies also reported that IMRT improved PTV coverage and/or OAR sparing [3, 5–7]. However, IMRT needs more MUs and longer treatment delivery time than 2D-RT or 3D-CRT, and it has inevitable disadvantages. Wang et al. evaluated the impact of prolonged treatment delivery time on tumor control in a biologic model, and calculated that cell killing was decreased with prolonged treatment delivery time, especially in tumors with low alpha/beta ratios or short repair half-times [12]. Although there are no clinical studies to date, several studies have shown the detrimental effect of prolonged treatment delivery time using cell lines or xenograft models [13–17].

VMAT allows rapid delivery of inverse planned radiotherapy by continuous gantry rotation and simultaneous modulation of MLCs, and has recently become available. Several studies have compared VMAT with fixed beam IMRT in terms of dose coverage, OAR-sparing effects, reduced treatment delivery time and decreased MUs [8–11]. In these studies, VMAT showed equivalent or better PTV coverage and OAR-sparing effects, decreased treatment delivery time and fewer MUs [8–11].

Studies of VMAT have been conducted for nasal cavity and paranasal sinus cancers, which are usually located between both eyes and have complex shaped target volumes [18–21]. The dosimetric superiority of VMAT for PTV coverage and OAR sparing shown in the present study are consistent with most previous studies. Sankaralingam et al. compared VMAT (two-arc) and coplanar IMRT (5-beam, sagittal) in nasal cavity and paranasal sinus cancer (n = 5) and reported that VMAT showed better homogeneity in all patients and better conformity in 3 of 5 patients. However, the number of patients was too small to establish statistically significant differences [19]. In another study, step-and-shoot IMRT was compared with single arc and multiple arc VMAT for localized prostate cancers (n = 5), pharyngeal cancers (n = 10), and paranasal sinus cancers (n = 5) [18]. VMAT showed equivalent or improved PTV coverage, homogeneity, conformity, and OAR sparing when compared with step-and-shoot IMRT in localized prostate cancers and pharyngeal cancers, but not in paranasal sinus cancer patients, where increased doses to the lenses were observed in VMAT using multiple arcs. More recently, Nguyen et al. reported a dosimetric comparative study which compared VMAT (3-arc) and IMRT (9-beam) for nasal cavity cancer (n = 10) [20]. In that study, VMAT showed comparable PTV coverage and equivalent or better reduced dose to the OARs as well as fewer MUs and shorter treatment delivery times. Similarly, VMAT showed equivalent PTV coverage to IMRT with decreased delivery times and MUs in 20 patients with head and neck cancers [21]. It is difficult to compare these studies directly because of differences in the target volumes, prescribed doses, number of prescribed dose levels, planning algorithm, plan evaluation methods, and small numbers of patients. However, VMAT shows better or equivalent PTV coverage and OAR-sparing effects in all studies except that of Guckenberger et al.

VMAT has greater freedom for optimization when compared with IMRT, as it allows gantry rotation with variable speed during treatment delivery, simultaneous MLC modification and simultaneous dose rate variation [22–25]. However, in nasal cavity and paranasal sinus cancer patients, whose target volumes are usually located between the eyes, the benefits of gantry rotation may be limited due to the relative limitations on beam angle selection. This could increase low-dose ‘tails’ to the lenses, and may account for the observations in Guckenberger et al. [18]. The present study evaluated whether the optimization freedom benefits of VMAT were realizable in nasal cavity or paranasal sinus cancers, or whether non-coplanar IMRT remained superior for PTV coverage and OAR sparing in that area. Three non-coplanar IMRT (7-, 11-, and 15-beam) plans were implemented for each patient, none of which were superior to VMAT in PTV coverage. All three IMRT plans had greater D_max_ and D_mean_, and worse mean DVH, for both lenses. Non-coplanar, multi-beam fixed beam IMRT may not therefore overrule the optimization freedom of VMAT, even if the number and angle of beams are optimal. Moreover, in the present study, beams from the posterior direction were the most limited because of the brainstem and brain. The superiority of the VMAT plan might also arise from this limitation of the posterior beams in the non-coplanar IMRT plans, but further studies using more posterior direction beams are needed to confirm this suggestion.

This study has several limitations. First, it is a planning study comparing the dosimetric parameters between non-coplanar IMRT and VMAT. Local control and toxicities can be influenced by various factors such as past medical history, histologic types, and combination with chemotherapy as well as by dosimetric parameters. Whether the superiority of VMAT can be translated into clinical benefit is therefore not clear. Several studies have reported that dosimetric improvement from 2D-RT or 3D-CRT to IMRT resulted in clinical benefit with respect to local control and toxicities, and technical advances within IMRT may also result in clinical improvements [7, 26–28]. However, the relative dosimetric superiority of VMAT to IMRT may be smaller than that of IMRT to two- or three-dimensional radiotherapy, and may not result in clinical difference. Moreover, in present planning study, there is also a general weakness that results may not be consistent when the calculation and/or optimization algorithms are changed. Second, the two-arc approach was used for VMAT planning whereas 7-, 11-, and 15-beam IMRT was planned. A single arc VMAT plan can be sufficient for areas with less complex shapes and single-dose prescription levels [18, 29–31]. However, for complex target areas or multiple dose prescription levels, the two-arc technique was needed to achieve PTV coverage and OAR sparing better than or equivalent to fixed beam IMRT, and single arc VMAT was inferior to fixed beam IMRT [10, 11, 18, 32, 33]. Nasal cavity and paranasal sinus cancers have complex target volume shapes, and so two-arc VMAT was chosen for the current study. Two-arc VMAT was equivalent to or better than non-coplanar IMRT in this setting. However, further studies of the details of VMAT such as the gantry rotation angle, number of arcs, and using non-coplanar arcs are needed. Third, the current study used a single-dose prescription level. The differences between VMAT and non-coplanar IMRT when performing simultaneous integrated boost techniques with multiple dose prescription level was not evaluated, and further studies are needed. Fourth, present study did not reflect the difference in the treatment delivery error between VMAT and non-coplanar IMRT. Although there has been no definite conclusion about the influence of delivery error yet, random and systematic errors of MLC position, gantry position, and MU are present in both VMAT and non-coplanar IMRT plans. Furthermore, couch rotation errors and intrafraction setup errors are also present in non-coplanar IMRT plans. Despite these shortcomings, the present study is one of a small number comparing VMAT with IMRT for nasal cavity and paranasal sinus cancers, and was performed in a relatively large number of patients. Attempts were made to reduce bias in several ways. First, all patients were re-planned for VMAT and non-coplanar IMRT, instead of using previous plans. Second, the same planning objectives were applied for VMAT and non-coplanar IMRT. Third, dose calculations were performed using the Anisotropic Analytic Algorithm. Furthermore, the MLC with 2.5 mm width in the Varian TrueBeam STx apparatus used in this study enables more precise plans for the complex shaped target volumes of nasal cavity and paranasal sinus cancers.

## Conclusions

In patients with nasal cavity or paranasal sinus cancer, VMAT provided better homogeneity and conformity for PTV than 7-, 11-, and 15- beam non-coplanar IMRT plans, while achieving equal or better OAR-sparing effects, using fewer MUs and shortening treatment delivery times. VMAT can therefore be considered a good treatment option for nasal cavity and paranasal sinus cancers.

## Abbreviations

VMAT: Volumetric modulated arc therapy
IMRT: Intensity modulated radiotherapy
PTV: Planning target volume
OAR: Organ at risk
D_max_: Maximum dose
D_mean_: Mean dose
HI: Heterogeneity index
CI: Conformity index
MUs: Monitor units
LINAC: Linear accelerator
CT: Computed tomography
GTV: Gross tumor volume
MRI: Magnetic resonance imaging
PET: Positron emission tomography
CTV: Clinical target volumes
MLCs: Multi-leaf collimators
D_5%_: Minimum doses delivered to 5% volume of the PTV
D_95%_: Minimum doses delivered to 95% volume of the PTV
DVHs: Dose volume histograms
2D-RT: Two-dimensional radiotherapy
3D-CRT: Three-dimensional conformal radiotherapy.
